# Supplementary material for: Early Outcomes of Right Ventricular Pressure and Volume Overload in an Ovine Model
Source: Biology (Basel). 2025 Feb 7;14(2):170. doi: 10.3390/biology14020170 (PMC11851871; doi:10.3390/biology14020170)
Supplement: Supplementary file 1 [file biology-14-00170-s001.zip › biology-3316678-supplementary.pdf]

Table S1. Anesthetic and emergency medications and their concentrations.

Emergency Drugs	
**Drug**	**Concentration**
**Atropine**	0.1 mg/mL
**Adrenaline**	0.1 mg/mL; 0.01 mg/mL; 0.001 mg/mL
**Ephedrine**	5 mg/mL
**1% Lidocaine**	10 mg/mL
**Metoprolol**	1 mg/mL
**Amiodarone**	50 mg/mL
**Anesthetic Drugs**	
**Drug**	**Concentration**
**Medetomidine**	1 mg/mL
**Midazolam**	1 mg/mL
**Propofol**	10 mg/mL
**Ketamine**	10 mg/mL
**Atracurium**	10 mg/mL

Table S2. Doses and routes of administration of medications used at different stages of anesthesia.

Drug	Dose	Route of Administration
**Pre-medication**		
**Medetomidine**	0.015 mg/kg	i.m
**Atropine**	0.01 mg/kg	s.c
**Induction**		
**Atropine**	0.01 mg/kg	i.v
**Midazolam**	0.05 mg/kg	i.v
**Ketamine**	1 mg/kg	i.v
**Propofol**	1 mg/kg	i.v
**Atracurium**	1 mg/kg	i.v
**Ceftriaxone**	30 mg/kg	i.v
**Maintenance**		
**Sevoflurane**	1.5–2.5%	inhalation
**Ketamine**	0.5 mg/kg	i.v
**Atracurium**	0.15 mg/kg	i.v
**Before anesthesia reversal**		
**Paracetamol**	10 mg/kg	i.v
**Ketorolac**	0.75 mg/kg	i.v
**Dexamethasone**	0.2 mg/kg	i.v
**Metoclopramide**	0.2 mg/kg	i.v
**Furosemide**	5–10 mg, as needed	i.v

Table S3. Monitoring protocol during the first 7 postoperative days.

General Condition	Good	Influenced	Poor
**Rectal temperature**			
**Neurological System**			
**Consciousness**	Conscious, alert	Altered consciousness	Unconscious
**Behavior**	Normal behavior	Lethargic, apathetic	Isolated
**Cardiovascular System**			
**Heart rate**			
**Jugular vein distension**	Present	Absent	
**Pulmonary System**			
**SpO_2**			
**Respiratory rate**			
**Auscultation**			
**Cough**	Present	Absent	
**Renal System**			
**Diuresis**	Present	Absent	
**Digestive System**			
**Rumination**	Present	Diminished	Absent
**Appetite**	Good	Diminished	Poor
**Stool**	Normal	Constipation	Diarrhea
**Meteorism**	Present	Absent	
**Teguments**			
**Edema**	Brisket edema	Submandibular edema	Absent
**Surgical incision aspect**	Healing	Dehiscent	Infected
**Drainage (mL)**			

Table S4. Postoperative treatment in the first 7 days.

Drug	Dose	Route of Administration	Administration Frequency
**Ceftriaxone**	30 mg/kg/dose	i.v	Twice daily
**Ceftiofur**	1 mg/kg/dose	i.m	Once daily
**Metronidazole**	15 mg/kg/dose	i.v	Twice daily
**Fluconazole**	6 mg/kg/dose	i.v	Every three days
**Furosemide**	5–10 mg	i.v	Once daily
	10–20 mg	p.o	Once daily
**Tramadol**	1 mg/kg/dose	s.c	Once–twice daily
**Metamizole**	10 mg/kg/dose	i.v	Once daily
**Paracetamol**	10 mg/kg/dose	i.v	Once–twice daily
**Dexamethasone**	0.5 mg/kg/day	i.v	Twice daily
**Spironolactone**	1 mg/kg/dose	p.o	Once–twice daily
**Potassium 40 mg**	1–2 tablets	p.o	Once–three times daily
**Simethicone 140 mg**	1–2 tablet	p.o	Once–twice daily
**Acetazolamide**	5 mg/kg/dose	p.o	Twice daily
**Ibuprofen**	10 mg/kg/dose	p.o	Twice daily

Table S5. Demographic data.

Variables	PAB (n = 6)	Annulotomy + TAP (n = 4)	Pulmonary Leaflet Perforation (n = 4)	
	Mean ± SD (Median)	Mean ± SD (Median)	Mean ± SD (Median)	**p-value**
**Age (weeks)**	14.4 ± 2.30 (14.0)	14.6 ± 1.3 (14.8)	13.0 ± 1.1 (12.8)	Ns
**Weight (kg)**	34.9 ± 12.70 (36.0)	34.6 ± 6.8 (36.5)	25.4 ± 8.8 (22.2)	Ns
**Height (cm)**	66.0 ± 14.90 (69.5)	69.8 ± 4.5 (70.0)	58.3 ± 17.4 (62.5)	Ns
**BSA (m^2)**	0.7 ± 0.2 (0.7)	0.7 ± 0.04 (0.7)	0.54 ±0.2 (0.5)	Ns

PAB—pulmonary artery banding; TAP—transannular patching; BSA—body surface area; SD—standard deviation.

Table S6. Hemodynamic parameters measured through Swan-Ganz catheterization.

Variables	PAB (n = 6) Preoperative Mean ± SD (Median)	PAB (n = 6) Postoperative Mean ± SD (Median)	p-value	Annulotomy + TAP (n = 4) Preoperative Mean ± SD (Median)	Annulotomy + TAP (n = 4) Postoperative Mean ± SD (Median)	p-value	Pulmonary Leaflet Perforation (n = 4) Preoperative Mean ± SD (Median)	Pulmonary Leaflet Perforation (n = 4) Postoperative Mean ± SD (Median)	p-value
**Swan-Ganz Hemodynamics**									
**sAP (mmHg)**	109.2 ± 20.1 (111.0)	88.7 ± 12.0 (89.5)	ns	116.5 ± 19.7 (117.0)	102.3 ± 13.2 (101.0)	Ns	108.8 ± 5.9 (108.0)	106.8 ± 14.5 (101.0)	Ns
**dAP (mmHg)**	82.7 ± 16.7 (90.0)	63.7 ± 13.5 (63.0)	0.0625	82.0 ± 21.8 (79.5)	68.8 ± 11.4 (71.5)	Ns	78.5 ± 6.0 (77.0)	75.5 ± 11.5 (77.0)	Ns
**mAP (mmHg)**	91.7 ± 17.5 (97.5)	72.0 ± 12.8 (72.0)	Ns	93.3 ± 20.9 (92.0)	80.0 ± 11.5 (81.5)	Ns	88.8 ± 5.5 (86.0)	85.5 ± 11.7 (84.5)	Ns
**HR (beats/min)**	109.2 ± 24.7 (106.5)	103.0 ± 16.1 (97.5)	Ns	99.3 ± 19.4 (94.0)	101.0 ± 5.0 (102.0)	Ns	108.3 ± 16.2 (105.5)	100.3 ± 5.7 (100.5)	Ns
**CVP (mmHg)**	6.8 ± 1.6 (7.0)	9.0 ± 4.1 (9.5)	Ns	5.8 ± 2.6 (6.0)	9.8 ± 2.1 (10.0)	Ns	5.3 ± 4.0 (5.0)	6.8 ± 4.1 (6.0)	Ns
**sRVP (mmHg)**	24.5 ± 6.5 (24.5)	53.5 ± 10.7 (49.5)	**0.0312**	27.3 ± 1.0 (27.5)	30.0 ± 2.2 (29.5)	Ns	26.8 ± 4.6 (28.5)	30.0 ± 5.7 (30.5)	Ns
**dRVP (mmHg)**	4.0 ± 4.0 (2.5)	12.3 ± 7.4 (12.0)	**0.0312**	4.8 ± 2.8 (4.5)	9.3 ± 2.5 (8.0)	Ns	5.8 ± 1.7 (5.5)	7.8 ± 2.5 (7.5)	Ns
**mRVP (mmHg)**	10.5 ± 3.9 (11.0)	25.8 ± 6.8 (25.0)	**0.0312**	12.3 ± 1.5 (12.0)	16.3 ± 1.9 (15.5)	Ns	12.8 ± 2.2 (13.0)	15.0 ± 2.9 (14.5)	Ns
**sPAP (mmHg)**	23.7 ± 5.3 (26.0)	18.3 ± 3.6 (19.5)	**0.0312**	25.8 ± 3.6 (26.5)	27.8 ± 1.5 (28.0)	Ns	25.8 ± 4.7 (27.5)	26.8 ± 5.8 (27.5)	Ns
**dPAP (mmHg)**	14.5 ± 4.6 (16.0)	10.2 ± 5.2 (11.5)	0.0625	17.5 ± 3.3 (18.0)	16.0 ± 2.9 (16.0)	Ns	16.0 ± 2.9 (15.5)	15.0 ± 3.9 (14.5)	Ns
**mPAP (mmHg)**	17.8 ± 4.8 (19.5)	13.0 ± 3.9 (14.0)	**0.0312**	20.5 ± 3.4 (21.0)	19.8 ± 2.6 (20.0)	Ns	19.5 ± 3.3 (20.0)	18.8 ± 4.6 (19.0)	Ns
**PAWP (mmHg)**	8.2 ± 1.9 (8.5)	8.8 ± 3.9 (9.5)	Ns	9.8 ± 2.6 (10.5)	12.0 ± 2.6 (12.0)	Ns	9.3 ± 2.1 (9.0)	9.8 ± 2.4 (9.0)	Ns

**SVI (mL/m²)**	61.0 ± 15.8 (67.4)	37.1 ± 12.8 (31.6)	**0.0312**	47.3 ± 9.8 (50.0)	58.6 ± 15.5 (62.9)	Ns	51.2 ± 19.1 (53.8)	63.2 ± 10.4 (67.0)	Ns
**CI (L/min/m²)**	6.6 ± 2.0 (6.4)	3.8 ± 1.4 (3.4)	**0.0312**	4.8 ± 1.7 (4.9)	5.9 ± 1.6 (6.1)	Ns	5.6 ± 2.3 (6.4)	6.3 ± 1.2 (6.6)	Ns
**SVRI (dynes·sec·cm⁻⁵·m²)**	1106.0 ± 340.0 (1165.0)	1401.0 ± 535.0 (1334.0)	Ns	1589.0 ± 626.0 (1318.0)	1050.0 ± 530.0 (831.0)	Ns	1455.0 ± 922.0 (1043.0)	1020.0 ± 210.0 (1013.0)	Ns
**PVRI (dynes·sec·cm⁻⁵·m²)**	120 ± 36.2 (121.5)	91.7 ± 33.6 (83.5)	**0.0312**	191.0 ± 112.0 (157.0)	113.3 ± 52.6 (108.0)	Ns	180 ± 132 (142.5)	117.3 ± 51.6 (123.0)	Ns
**LCWI (kg·m/m²)**	7.6 ± 2.7 (7.7)	3.3 ± 1.3 (3.3)	**0.0312**	5.7 ± 3.1 (4.9)	5.3 ± 1.1 (5.3)	Ns	6.1 ± 2.8 (6.5)	6.6 ± 1.8 (6.3)	Ns
**LVSWI (g·m/m²)**	69.7 ± 24.7 (65.5)	31.7 ± 11.5 (32.4)	**0.0312**	54.2 ± 19.1 (51.1)	52.5 ± 9.7 (53.6)	Ns	55.6 ± 21.8 (60.3)	65.4 ± 15.5 (65.0)	Ns
**RCWI (kg·m/m²)**	1.0 ± 0.5 (1.0)	0.2 ± 0.1 (0.2)	**0.0312**	0.9 ± 0.3 (1.1)	0.8 ± 0.4 (0.8)	Ns	1.0 ± 0.7 (0.8)	1.0 ± 0.4 (0.9)	Ns
**RVSWI (g·m/m²)**	9.2 ± 4.5 (8.6)	2.0 ± 0.7 (1.9)	**0.0312**	9.5 ± 3.4 (9.6)	8.1 ± 3.6 (7.8)	Ns	9.2 ± 4.3 (7.3)	10.2 ± 3.4 (9.2)	Ns

PAB—pulmonary artery banding; TAP—transannular patching; sAP—systolic arterial pressure; dAP—diastolic arterial pressure; mAP—mean arterial pressure; HR—heart rate; CVP—central venous pressure; sRVP—systolic right ventricular pressure; dRVP—diastolic right ventricular pressure; mRVP—mean right ventricular pressure; sPAP—systolic pulmonary artery pressure; dPAP—diastolic pulmonary artery pressure; mPAP—mean pulmonary artery pressure; PAWP—pulmonary artery wedge pressure; SVI—stroke volume index; CI—cardiac index; SVRI—systemic vascular resistance index; PVRI—pulmonary vascular resistance index; LCWI—left cardiac work index; LVSWI—left ventricular stroke work index; RCWI—right cardiac work index; RVSWI—right ventricular stroke work index.

Table S7. Parameters measured through arterial blood gas analyses.

Variables	PAB (n = 6)			Annulotomy + TAP (n = 4)			Pulmonary Leaflet Perforation (n = 4)		
	**Preoperative** Mean ± SD (Median)	**Postoperative** Mean ± SD (Median)	**p-value**	**Preoperative** Mean ± SD (Median)	**Postoperative** Mean ± SD (Median)	**p-value**	**Preoperative** Mean ± SD (Median)	**Postoperative** Mean ± SD (Median)	**p-value**
**Arterial Blood Gas Analyses**									
**$ScvO_2$ (%)**	89.2 ± 8.8 (91.5)	79.5 ± 10.7 (83.0)	**0.0312**	86.3 ± 4.6 (85.5)	84.0 ± 3.5 (85.0)	Ns	87.8 ±5.7 (87.5)	89.0 ± 4.6 (89.0)	Ns
**pH**	7.5 ± 0.1 (7.4)	7.4 ± 0.1 (7.4)	Ns	7.4 ± 0.04 (7.4)	7.4 ± 0.05 (7.4)	Ns	7.4 ± 0.05 (7.4)	7.4 ± 0.1 (7.4)	Ns
**pO_2 (mmHg)**	183.8 ± 42.1 (199.5)	147.8 ± 53.5 (158.0)	Ns	201.3 ±123.4 (148.5)	126.0 ± 62.2 (102.5)	Ns	182.3 ± 31.3 (189.5)	186.3 ± 29.8 (182.5)	Ns
**pCO_2 (mmHg)**	43.9 ± 13.8 (46.1)	41.4 ± 14.9 (42.8)	Ns	48.0 ± 6.4 (47.1)	47.8 ± 3.4 (47.8)	Ns	48.3 ± 5.6 (49.3)	49.2 ± 4.1 (50.0)	Ns
**SaO_2 (%)**	99.7 ± 0.5 (100.0)	97.3 ± 5.6 (99.5)	Ns	99.5 ± 0.6 (99.5)	97.3 ± 2.5 (97.5)	Ns	99.8 ± 0.5 (100.0)	99.5 ± 0.6 (99.5)	Ns
**HCO_3^- (mmol/L)**	29.1 ± 3.9 (28.4)	26.5 ± 3.9 (27.4)	Ns	29.4 ± 4.5 (27.7)	27.5 ± 3.1 (27.7)	Ns	30.5 ± 2.5 (30.5)	29.7 ± 2.1 (30.6)	Ns
**BE (mmol/L)**	5.0 ± 3.3 (4.0)	2.3 ± 2.3 (3.0)	Ns	4.5 ± 5.3 (3.0)	2.0 ± 4.1 (2.0)	Ns	6.0 ± 2.9 (5.5)	4.3 ± 3.6 (5.5)	Ns
**Lactate (mmol/L)**	0.5 ± 0.3 (0.4)	1.4 ± 0.9 (1.0)	**0.0312**	0.4 ± 0.06 (0.3)	0.9 ± 0.6 (0.7)	Ns	0.5 ± 0.3 (0.4)	0.6 ± 0.4 (0.5)	Ns
**Hb (g/dL)**	11.9 ± 1.3 (11.8)	11.3 ± 1.1 (11.4)	Ns	11.9 ± 1.3 (12.0)	11.6 ± 0.7 (11.5)	Ns	11.7 ± 0.8 (11.4)	10.8 ± 0.3 (10.8)	Ns
**Hct (%)**	35.3 ± 3.8 (35.0)	33.5 ± 3.3 (33.5)	Ns	35.5 ±4.2 (35.5)	35.0 ± 2.5 (35.0)	Ns	35.0 ± 2.2 (34.5)	32.3 ± 1.0 (32.5)	Ns
**Na^+ (mmol/L)**	152.3 ± 1.2 (152.5)	151.7 ± 2.3 (151.5)	Ns	146.8 ± 1.3 (147.0)	144.8 ± 2.5 (144.5)	Ns	150.0 ± 5.0 (148.5)	146.8 ± 3.2 (146.5)	Ns
**K^+ (mmol/L)**	3.4 ± 0.4 (3.3)	3.6 ± 0.2 (3.5)	Ns	3.2 ± 0.5 (3.3)	3.5 ± 0.4 (3.4)	Ns	3.3 ± 0.2 (3.3)	3.2 ± 0.5 (3.1)	Ns

$ScvO_2$—central venous oxygen saturation; pO_2—oxygen partial pressure; pCO_2—carbon dioxide partial pressure; SaO_2—arterial oxygen saturation; HCO_3^-—bicarbonate; BE—base excess; Hb—hemoglobin; HCT—hematocrit; Na^+—sodium; K^+—potassium.

Table S8. Total chest drainage period and quantity.

	Surgical Procedure Type		
	**Annulotomy + TAP (*n* = 4)**	**Pulmonary Leaflet Perforation (*n* = 4)**	**PAB (*n* = 6)**
**Number of days with chest tube (mean)**	3.25	2.25	2.4
**Total drainage in mL (mean)**	890	380	588
